# Evaluation of Mandibular Fixation Techniques Using Monocortical Plates After Mandibular Setback Surgery

**DOI:** 10.3390/life15060845

**Published:** 2025-05-23

**Authors:** Seung-Woo Lee, Bong-Jin Jeong, Junho Jung

**Affiliations:** 1 Department of Oral & Maxillofacial Surgery, Kyung Hee University College of Dentistry, Kyung Hee University Medical Center, Seoul 02447, Republic of Korea; 2 Department of Dentistry, Graduate School, Kyung Hee University, Seoul 02447, Republic of Korea

**Keywords:** orthognathic surgery, sagittal split ramus osteotomy, fixation method, plate and screw, surgical relapse

## Abstract

This study aimed to evaluate mandibular fixation techniques using monocortical plates following sagittal split ramus osteotomy in skeletal Class III patients. Ninety-three patients were categorized into three groups based on fixation methods: four-hole miniplate with one proximal and two distal screws (Group 1); four-hole miniplate with four screws (Group 2); sliding plate with two proximal and one distal screws (Group 3). Cone-beam computed tomography scans were obtained at three time points: immediately postoperative (T1), 6 months (T2), and 12 months (T3). The yaw, roll, and pitch rotations of the proximal segment, as well as horizontal and vertical changes of the pogonion, were evaluated. Group 1 exhibited significantly greater counterclockwise rotation of the proximal segments at T2 (*p* = 0.021) and T3 (*p* = 0.035) compared to the other groups. Additionally, Group 1 showed significantly smaller anterior and superior displacement of the pogonion at T3 (0.97 ± 2.10 mm, *p* = 0.009; 0.03 ± 1.62 mm, *p* = 0.011, respectively). Following surgical wafer removal, intimate occlusal contact is archived and the elimination of premature contacts through postoperative orthodontic treatment contributes to counterclockwise autorotation of the mandible. Therefore, anterior and superior movements of the pogonion are expected if firm fixation between the proximal and distal segments is achieved. Therefore, these findings suggest that a single proximal screw, as seen in a three-screw fixation, may act as a fulcrum, insufficiently resisting postoperative clockwise rotation of the distal segments.

## 1. Introduction

Postoperative stability remains a primary concern in orthognathic surgery to ensure optimal functional and aesthetic outcomes. Various techniques have been developed to minimize the displacement of the proximal and distal mandibular segments. Rigid fixation using multiple bicortical screws has been implemented to achieve stable intersegmental fixation. This method offers several advantages, including stable outcomes with minimal need for postoperative intermaxillary fixation, early recovery of mandibular function, and reduced risk of postoperative relapse [1,2]. However, the use of bicortical screws can induce undesirable positional changes originated from difficulty in maintaining intersegmental gaps, resulting in the rotation of the condyle, potentially compromising condylar change and skeletal stability [3,4,5].

Semirigid fixation has emerged as an alternative approach, providing adequate stabilization of osteotomized segments while allowing flexibility to support bone healing. Monocortical miniplates, in particular, offer advantages, such as avoiding extraoral incisions and reducing the risk of injury to the inferior alveolar nerve. They can also be easily contoured to adapt to bony segments and repositioned if necessary [3]. Although a monocortical plate may not be as rigid as a bicortical plate, its clinical usefulness and outcomes in orthognathic surgery have been proven acceptable in several studies [6,7,8,9]. While using four screws in a four-hole miniplate can provide more stability for the bony segments [10], some advocate for using three screws in a four-hole miniplate or three-hole sliding plate to allow minor condylar movement [11,12,13]. One study suggested that placing one screw in a four-hole miniplate at the proximal segment may provide flexibility for adjusting the condylar position through physiological muscle activity postoperatively and the postoperative relapse did not differ from that with the four-screw fixation [11]. Semirigid three-hole sliding plates have also been proposed as a viable fixation method [12,14]. The oval-shaped sliding slot in the distal portion allows for controlled movement of the proximal segment following bilateral sagittal split ramus osteotomy (BRSSO), providing physiologic condylar movement and potentially minimizing early skeletal relapse [12,14,15]. Although it also uses three screws in a miniplate, the difference from the previous study [11] is that one screw in a bony segment is located in the distal portion. The distance between the rotational axis, determined by one screw, and the pterygomasseteric sling differs, which may significantly affect the postoperative rotation of the proximal segment and potential relapse.

A recent systematic review and meta-analysis reported no statistically significant difference in postoperative stability between bicortical screw fixation and miniplate fixation following mandibular setback using BSSRO [16]. However, previous studies comparing sliding plates with conventional four-hole miniplates have been limited by small sample sizes, reducing the generalizability of their findings [13,14,17,18,19]. Furthermore, few studies to date have directly compared different miniplate configurations or examined the influence of varying screw numbers on postoperative stability. Additionally, no studies have compared them with sliding plates, which allow for physiological movement and could potentially reduce postoperative relapse. Therefore, the present study aimed to assess positional changes in the mandibular segments using a large sample size, comparing three semirigid fixation methods: a conventional miniplate with three monocortical screws (one proximal and two distal screws), four monocortical screws, and a monocortical sliding plate.

## 2. Materials and Methods

### 2.1. Subjects

Patients diagnosed with mandibular prognathism who underwent BSSRO with or without concurrent Le Fort I osteotomy at the Department of Oral and Maxillofacial Surgery of the K University Medical Center between January 2013 and October 2020 were selected. Patients who met the following criteria were included: (1) those who were aged ≥ 19 years, (2) those who had periodic follow-up radiographic data (immediate postoperatively, 6 months postoperatively, 12 months postoperatively), (3) those with a discrepancy of less than 5 mm between the left and right setback amounts, as measured at the mental foramen. The following patients were excluded: (1) those who were accompanied by genioplasty; (2) those who presented with craniofacial anomalies; (3) those who had a history of metabolic bone diseases and maxillofacial trauma; (4) those who underwent any revision surgery after the initial orthognathic surgery. The fixation techniques were determined according to each surgeon’s preference and were not based on any patient parameters or clinical criteria. The present study was approved by the Ethics Committee of Kyung Hee University Medical Center (KH-DT22022).

**Figure 1:**
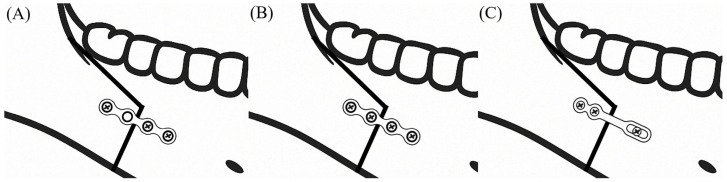
Three different fixation methods used in this study. (**A**) Group 1: 4-hole monocortical miniplate with 3 screws (1 screw placed in the proximal segment and 2 screws in the distal segment); (**B**) Group 2: 4-hole monocortical miniplate with 4 screws; (**C**) Group 3: sliding plate with 2 screws placed in the proximal segment and 1 screw in the distal segment.

A total of 93 patients (51 males and 42 females; mean age, 23.3 years) were included in this study (Table 1) and they were divided into three groups based on the three fixation methods as follows (Figure 1):

Group 1 (3 screws): 1 conventional 4-hole monocortical miniplate with 1 screw placed in the proximal segment and 2 screws in the distal segment (Jeil Medical Corp., Seoul, Republic of Korea);

Group 2 (4 screws): 1 conventional 4-hole monocortical miniplate with 2 screws placed in both the proximal and distal segments (Jeil Medical Corp.);

Group 3 (sliding plate): 1 3-hole monocortical sliding plate with 2 screws placed in the proximal segment and 1 screw in the distal segment (Jeil Medical Corp.).

Group 1 included 25 patients (12 males and 13 females) with a mean age of 24.5 ± 7.0 years. Group 2 comprised 40 patients (22 males and 18 females) with a mean age of 22.7 ± 4.9 years. Group 3 consisted of 28 patients (17 males and 11 females) with a mean age of 22.9 ± 4.8 years. The amounts of mandibular setback for each group were 4.8 mm, 5.6 mm, and 5.6 mm, respectively (*p* = 0.095).

### 2.2. Preoperative Planning and Surgical Procedure

After consulting with orthodontists, who performed the presurgical orthodontic treatment, the amount and direction of movement for the maxilla and mandible, as well as postoperative occlusion, were determined. A virtual model surgery was conducted using computed tomography (CT) data and scanned dental casts.

Following a Le Fort I osteotomy and fixation of the maxilla using an intermediate occlusal wafer prepared presurgically, a sagittal split ramus osteotomy was performed to reposition the mandibular distal segment posteriorly. After removing bony interference between the proximal and distal segments, intermaxillary fixation was achieved using an occlusal wafer prepared as part of the surgical plan. To establish the centric relation of the proximal segment, the distance between the proximal segment and the maxillary dental brackets was measured before mandibular osteotomy and confirmed before the fixation of the distal and proximal segments, as previously described [20]. After intermaxillary fixation (IMF) with the occlusal wafer, the three different fixation methods were applied for the osteosynthesis of the bony segments. The IMF was maintained for 2 weeks postoperatively, followed by occlusal guidance using elastic rings for an additional 4 weeks.

### 2.3. Data Acquisition

Cone-beam computed tomography (CBCT) scans were performed at three time points: immediately postoperatively (T1), 6 months postoperatively (T2), and 12 months postoperatively (T3), using the Alphard Vega 3030 dental CT system (Asahi Roentgen Ind. Co., Ltd., Kyoto, Japan) with a standard protocol (80 kVp, 5 mA, 17 s of exposure, 0.3 mm of voxel size). All patients were imaged in an upright position for CBCT scans.

### 2.4. Measuring the Movements of the Proximal Segment and Relapse

CBCT data were analyzed using three-dimensional imaging software v1.0 (OnDemand 3D; Cybermed, Seoul, Republic of Korea). The midsagittal plane was defined as the plane passing through the nasion, sella, and the midpoint between the bilateral orbitales. The horizontal plane was established perpendicular to the midsagittal plane and passed through both orbitals and the right porion. The frontal plane was defined as perpendicular to both the midsagittal and horizontal planes.

**Figure 2:**
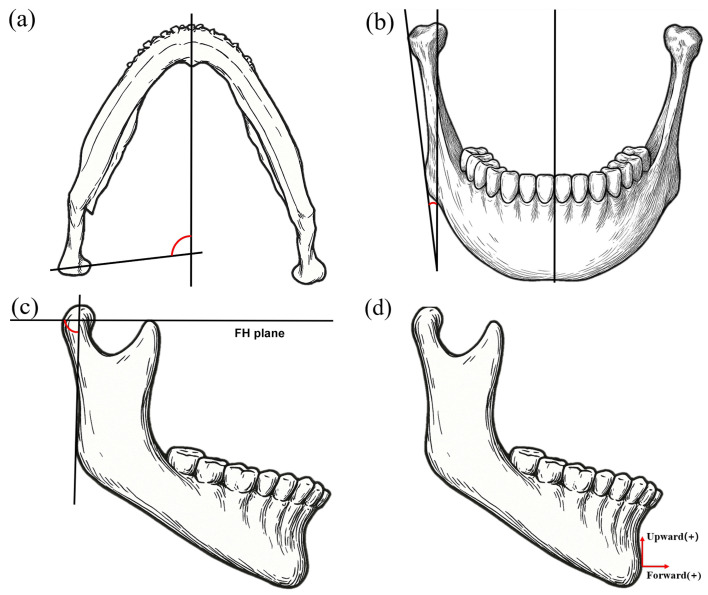
Measuring the displacement of the proximal segment and relapse; (**a**) yaw movement, (**b**) roll movement, (**c**) pitch movement, (**d**) relapse evaluation (the pogonion).

Yaw movement was assessed by measuring the angle between the long axis of the mandibular condylar head and the midsagittal plane in the axial view. Inward rotation of the proximal segment was assigned negative values, whereas outward rotation was assigned positive values (Figure 2a).

Roll movement was evaluated by measuring the frontal inclination of the ramus, defined as the angle between the horizontal plane and a line connecting the most lateral point of the condylar head and the posterior gonion in the frontal view. Medial rotation of the proximal segment was recorded as positive while lateral rotation was recorded as negative (Figure 2b).

Pitch movement was evaluated by measuring the sagittal inclination of the ramus, defined as the angle between the horizontal plane and a line connecting the most superior point of the condylar head and the posterior gonion in the sagittal view. Backward (clockwise) rotation of the proximal segment was assigned negative values while forward (counterclockwise) rotation was assigned positive values (Figure 2c).

To assess relapse of the distal segment, the anteroposterior (y-axis) and vertical (z-axis) coordinates of the pogonion were recorded and compared among the groups. In the horizontal plane, forward displacement was assigned positive values and backward displacement negative values. In the vertical plane, upward displacement was considered positive and downward displacement negative (Figure 2d).

All measurements in this study were conducted twice, with a two-week interval between assessments, to minimize intra-examiner error.

### 2.5. Statistical Analysis

Statistical analyses were performed using SPSS software (version 26.0; IBM Corp., Armonk, NY, USA). Chi-square tests and the Kruskal–Wallis test were used to assess demographic differences among the groups. Differences among the three groups at each time point were assessed using a one-way analysis of variance (ANOVA) followed by a Bonferroni post hoc test as the recorded data were normally distributed. To analyze time-course changes, repeated-measures ANOVA was utilized. The intraclass correlation coefficient (ICC) values ranged from 0.863 to 0.932.

## 3. Results

### 3.1. Postoperative Rotational Movement of the Proximal Segment

Table 2 and Figure 2 summarize changes in the proximal segment position, assessed by yaw, roll, and pitch relapses.

During the first 6 months postoperatively (T2–T1), the mean yaw movement was 1.66° in Group 1, 1.75° in Group 2, and 1.53° in Group 3. The total change over 12 months (T3–T1) indicated outward rotation in all groups, with mean values of 1.55°, 1.38°, and 2.22° for Groups 1, 2, and 3, respectively. Group 3 exhibited the greatest variation in yaw movement at 12 months. However, the differences were statistically insignificant among the groups (*p* = 0.404).

All groups exhibited medial roll movement of the proximal segment over the first six months (T2–T1). Group 1 displayed the most significant medial rotation at 2.24°, followed by Group 2 with 0.99°, and Group 3 with 0.80°. The total change over 12 months (T3–T1) also reflected medial rotation: 2.06° in Group 1, 1.31° in Group 2, and 0.78° in Group 3. Group 1 exhibited the largest variation in roll movement at 12 months. Nonetheless, no significant differences were found among the groups (*p* = 0.099).

In pitch movement, a significant difference among the groups was observed at both 6 months postoperatively (T2–T1, *p* = 0.021) and 12 months postoperatively (T3–T1, *p* = 0.035). The average pitch movement during the first 6 months (T2–T1) was 2.74° for Group 1, 1.74° for Group 2, and 1.89° for Group 3. The cumulative pitch movement over 12 months (T3–T1) was 2.86° in Group 1, 1.86° in Group 2, and 2.07° in Group 3. The statistical comparison revealed that counterclockwise rotation in Group 1 was significantly greater than in Group 2 at T2–T1 (*p* = 0.022) and at T3–T1 (*p* = 0.033, Table 2). However, there was no statistical significance between Groups 2 and 3. Most postoperative movements of the proximal segments occurred within the first six months after the operation. The changes observed after six months were not significantly different between the groups (*p* = 0.947).

### 3.2. Relapses of the Distal Segment

All groups demonstrated anterior movement of the pogonion during the first 6 months postoperatively (T2–T1), with mean values of 0.98 mm in Group 1, 1.61 mm in Group 2, and 1.99 mm in Group 3 (Table 3). The displacements that occurred between 6 and 12 months postoperatively (T3–T2, –0.01 mm in Group 1, 0.40 mm in Group 2, and 0.04 mm in Group 3) were relatively smaller than the ones that occurred before 6 months postoperatively. The total anterior displacement at 12 months postoperatively (T3–T1) was 0.97 mm for Group 1, 2.01 mm for Group 2, and 2.03 mm for Group 3. Group 1 exhibited significantly smaller horizontal pogonion movement than Group 3 during the first 6 months postoperatively (T2–T1; *p* = 0.012). Over the entire period (T3–T1), horizontal displacement in Group 1 was significantly smaller compared to Group 2 (*p* = 0.015) and Group 3 (*p* = 0.025). Additionally, significant differences in the time-course changes of horizontal displacement were observed among the three groups (*p* = 0.045).

The average vertical pogonion movement at the first 6 months (T2–T1) was 0.12 mm in Group 1, 1.05 mm in Group 2, and 1.03 mm in Group 3 (Table 3). Over the entire period (T3–T1), the pogonion moved upward by 0.03 mm in Group 1, 1.01 mm in Group 2, and 1.29 mm in Group 3. Statistical analysis indicated that vertical pogonion movement in Group 1 was significantly smaller than in Group 2 (*p* = 0.047) and Group 3 (*p* = 0.013). Significant differences in time-course changes were also noted in vertical displacement among the groups (*p* = 0.036).

## 4. Discussion

Ensuring postoperative stability is critical in mandibular setback surgery to achieve optimal outcomes and prevent skeletal relapse. Various factors influence the risk of postoperative relapse, including positional changes of the mandibular condyle, the magnitude and direction of segmental movement, fixation technique, masticatory muscle forces, and the nature of postoperative orthodontic treatment [21,22]. Numerous studies have evaluated different fixation methods in the context of BSSRO and mandibular setback, particularly with respect to their impact on postoperative stability and relapse rates. In a study comparing conventional four-hole miniplates with varying numbers of screws in the proximal segment, there was no statistically significant differences in stability [11]. However, prior studies examining the sliding plate versus conventional miniplates have often been limited by small sample sizes, which hinders the strength of their conclusions. [13,14,17,18,19]. Therefore, the present study aimed to address this limitation by analyzing postoperative intersegmental displacement and relapse among different semirigid fixation methods using a larger patient cohort.

Previous research has explored the direction and magnitude of proximal segment displacement in the correction of skeletal Class III malocclusion. Common observations immediately postoperatively include inward yaw rotation, lateral roll, and clockwise pitch rotation [23,24,25,26,27]. Over time, there tends to be a reversion of the segments toward their original positions. Previous research has examined the direction and magnitude of proximal segment displacement during the correction of skeletal Class III malocclusion. Inward yaw rotation, lateral roll [26], and clockwise pitch rotation [27] have commonly been observed preoperatively, with a tendency for the segments to revert toward their original position after surgery [11,28,29,30]. Our findings were consistent with these patterns. Notably, patients with three-screw fixations demonstrated significantly greater counterclockwise pitch rotations than patients with other fixation methods.

The pogonion has been utilized in several studies to evaluate surgical relapse after orthognathic surgery. It tended to shift forward in the horizontal direction and upward in the vertical direction following surgery [12,23,28,31]. The anterior and superior shift of the pogonion is not solely due to surgical relapse caused by intersegmental displacement. Counterclockwise rotation of the whole mandible occurs following removal of the surgical wafer due to its interocclusal thickness and close occlusal contact. Additionally, the elimination of premature occlusal contacts during postoperative orthodontic treatment may induce further counterclockwise rotation, leading to mandibular autorotation that mimics skeletal relapse. Therefore, anterior and superior movements of the pogonion are expected if firm fixation between the proximal and distal segments is achieved. In the present study, patients with a four-screw fixation and sliding plate demonstrated consistent postoperative positional changes, similar to those reported in other studies [14]. Notably, the three-screw fixation, which includes only one screw at the proximal segment, exhibited significantly smaller anterior and superior displacement. This may be due to compensation for the counterclockwise autorotation by the clockwise rotation of the distal segment. Therefore, although the anterior and superior displacement of the pogonion is smaller with a three-screw fixation, the risk and tendency for a postoperative open bite appear to be relatively higher than with other fixation methods.

In a study comparing sliding plates, four-hole miniplates, and bicortical screws, similar results were demonstrated [14]. Although there were no overall statistically significant differences among the three groups, bicortical fixation showed a significantly greater superior and anterior shift of the pogonion six months post-operation. It is well-known that bicortical fixation is relatively firm and allows for less postoperative relapse. Therefore, it is assumed that a similar pattern of distal segment movement occurred as in our study. This means that the counterclockwise rotation after the removal of the wafer and postoperative orthodontic treatment was compensated by the clockwise rotation of the distal segment in less-rigid fixation with sliding plates and miniplates. Another study also suggested that there were no significant changes between sliding plates and four-hole miniplates in the horizontal displacement of the distal segment [13].

**Figure 3:**
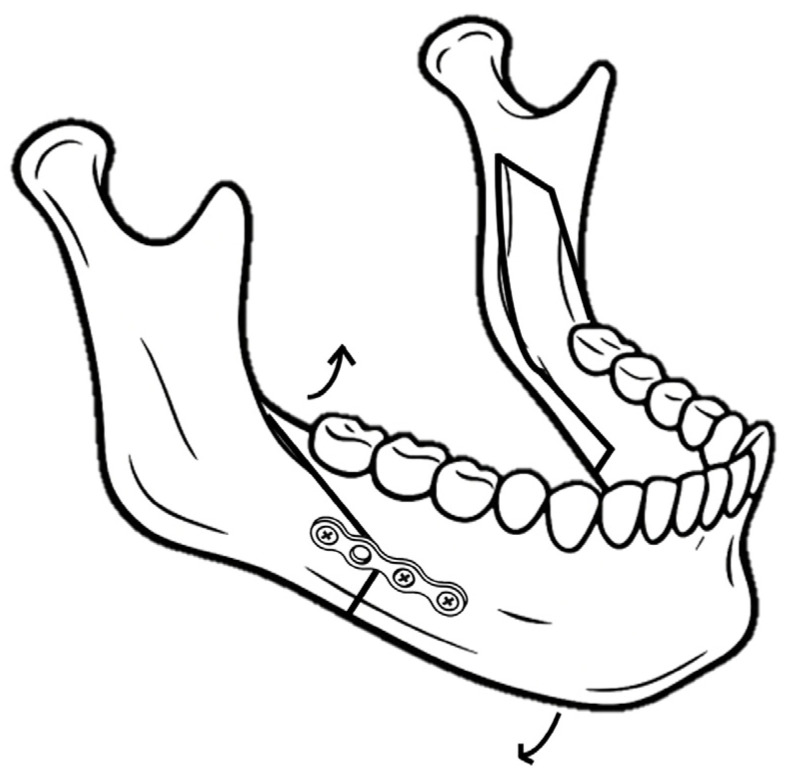
Relapse pattern on mandibular segments.

Postoperative counterclockwise rotation of the proximal segment was significantly higher in the patients with three-screw fixations. Based on this finding, it is assumed that a single proximal screw may act as a fulcrum, insufficiently resisting postoperative clockwise rotation of the distal segments (Figure 3). Due to the tension from pterygomasseteric sling and the temporalis, which tends to rotate the ramus forward and upward, the counterclockwise rotation of the proximal segment may occur. In contrast, suprahyoid muscle activity and occlusal forces exert posterior and inferior traction on the chin. These muscular forces may have a more pronounced effect in the less rigid three-screw configuration, potentially leading to deformation or fracture of the miniplate and undesirable outcomes, such as anterior open bite or chin deviation [32,33]. Overall, while relatively rigid fixation induces anterior and superior displacement of the chin due to autorotation from close interocclusal contact, three-screw fixation tends to result in a postoperative open bite.

The sliding plate demonstrated a similar pitch movement and displacement pattern of the distal segment to the four-screw fixation. The sliding plate design, which features an oval-shaped hole at the distal part, allows controlled movement between the proximal and distal segments, thereby compensating for early relapse from the mandibular condyle and minimizing the risk of temporomandibular joint complications [12,15]. Our findings also suggested that the displacement pattern of the chin is not inferior to more rigid fixation, which refers to the four-screw fixation. In a study investigating the stability of sliding plates, the pogonion moved in the anterior and superior directions approximately 1–2 mm after postoperative orthodontic treatment, similar to our results [12]. Considering that the sliding plate also uses three screws, this implies that the position of the screws is a crucial factor. The distance between the pterygomandibular sling and a single screw, which may act as a fulcrum, might be a key factor. Fixation with at least two screws in the proximal segment appears essential for postoperative stability.

Despite the relatively larger sample size compared to prior studies, this study still seems insufficient to draw definitive conclusions due to the retrospective nature of data collection. Furthermore, the numerical imbalance between the groups could potentially undermine the reliability of the statistical outcomes. Future research should include larger cohorts and employ randomized controlled trial designs to validate these findings. A larger cohort and multicenter center study may promise a firm and steady result for this aspect. In addition, applying a superimposition method for sequential CT images from different time points may enhance the reliability of this study by reducing measurement errors. Nevertheless, this study provides valuable clinical insights regarding fixation methods and their influence on postoperative stability following mandibular setback surgery.

## 5. Conclusions

Following surgical wafer removal, close occlusal contact between the maxillary and mandibular dentition is achieved and the elimination of premature contacts through postoperative orthodontic treatment contributes to counterclockwise autorotation of the whole mandible. Therefore, anterior and superior movements of the chin are expected if a firm fixation between the proximal and distal segments is achieved. Therefore, although three-screw configuration, in which only one screw was used in the proximal segment, exhibited minimal anterior and superior displacement of the distal segment, it is assumed that a single proximal screw may act as a fulcrum that insufficiently resists postoperative clockwise rotation of the distal segments and it may increase the risk and tendency for postoperative open bite. These findings suggest that dual-screw fixation in the proximal segment, as seen in four-screw fixation and sliding plates, may enhance postoperative stability and reduce the risk of undesirable mandibular displacement.

## Figures and Tables

**Table 1:** Demographic data.

Variable	Group 1	Group 2	Group 3	*p*-Value
Subject (male/female)	25 (12/13)	40 (22/18)	28 (17/11)	0.653
Age (years)	24.5 ± 7.0	22.7 ± 4.9	22.9 ± 4.8	0.431
Amount of mandibular setback (mm)	4.8	5.6	5.6	0.095

Group 1: four-hole monocortical miniplate with three screws (one screw placed in the proximal segment and two screws in the distal segment); Group 2: four-hole monocortical miniplate with four screws; Group 3: sliding plate with two screws placed in the proximal segment and one screw in the distal segment.

**Table 2:** Rotational movements of the proximal segment of the mandible.

	Group 1	Group 2	Group 3	*p*-Value
Yaw movement (°)				
T2–T1	1.66 (±4.17) ^a^	1.75 (±3.38) ^a^	1.53 (±3.30) ^a^	0.939
T3–T2	−0.11 (±2.65) ^a^	−0.37 (±2.90) ^a^	0.69 (±3.11) ^a^	0.105
T3–T1	1.55 (±4.08) ^a^	1.38 (±3.28) ^a^	2.22 (±3.77) ^a^	0.404
Roll movement (°)				
T2–T1	2.24 (±5.01) ^a^	0.99 (±1.75) ^a^	0.80 (±2.49) ^a^	0.039 *
T3–T2	−0.17 (±2.10) ^a^	0.32 (±1.40) ^a^	−0.02 (±2.25) ^a^	0.316
T3–T1	2.06 (±4.60) ^a^	1.31 (±1.86) ^a^	0.78 (±2.72) ^a^	0.099
Pitch movement (°)				
T2–T1	2.74 (±1.76) ^a^	1.74 (±1.98) ^b^	1.89 (±2.38) ^ab^	0.021 *
T3–T2	0.12 (±1.28) ^a^	0.12 (±1.18) ^a^	0.19 (±1.47) ^a^	0.947
T3–T1	2.86 (±1.78) ^a^	1.86 (±1.87) ^b^	2.07 (±2.78) ^ab^	0.035 *

Group 1: three screws; Group 2: four screws; Group 3: sliding plate. T1: immediately after surgery; T2: 6 months after surgery; T3: 1 year after surgery. *p* < 0.05 was considered statistically significant. Asterisks (*) indicate statistical significance between the groups. The same superscript letter indicates statistical insignificance and different letters indicate statistical significance.

**Table 3:** Relapses of the distal segment.

	Group 1	Group 2	Group 3	*p*-Value
Horizontal relapse (mm)				
T2–T1	0.98 (±1.56) ^a^	1.61 (±1.84) ^ab^	1.99 (±1.87) ^b^	0.015 *
T3–T2	−0.01 (±1.07) ^a^	0.40 (±0.92) ^a^	0.04 (±0.99) ^a^	0.034 *
T3–T1	0.97 (±2.10) ^a^	2.01 (±1.94) ^b^	2.03 (±2.10) ^b^	0.009 *
Vertical relapse (mm)				
T2–T1	0.12 (±1.55) ^a^	1.05 (±1.55) ^a^	1.03 (±1.69) ^a^	0.051
T3–T2	−0.09 (±0.97) ^a^	−0.04 (±0.87) ^a^	0.26 (±0.94) ^a^	0.304
T3–T1	0.03 (±1.62) ^a^	1.01 (±1.50) ^b^	1.29 (±1.61) ^b^	0.011 *

Group 1: three screws; Group 2: four screws; Group 3: sliding plate. T1: immediately after surgery; T2: 6 months after surgery; T3: 1 year after surgery. *p* < 0.05 was considered statistically significant. Asterisks (*) indicate statistical significance between the groups. The same superscript letter indicates statistical insignificance and different letters indicate statistical significance.

## Data Availability

The raw data supporting the conclusions of this article will be made available by the authors upon request.
